# Cost-effectiveness of expanded hepatitis A vaccination among adults with diagnosed HIV, United States

**DOI:** 10.1371/journal.pone.0282972

**Published:** 2023-03-17

**Authors:** Taiwo O. Abimbola, Michelle Van Handel, Yunfeng Tie, Lijing Ouyang, Noele Nelson, John Weiser

**Affiliations:** 1 National Center for HIV, Viral Hepatitis, STD and TB Prevention, Centers for Disease Control and Prevention, Atlanta, Georgia, United States of America; 2 Division of HIV Prevention, National Center for HIV, Viral Hepatitis, STD and TB Prevention, Centers for Disease Control and Prevention, Atlanta, Georgia, United States of America; 3 Division of Reproductive Health, National Center for Chronic Disease Prevention and Health Promotion, Centers for Disease Control and Prevention, Atlanta, Georgia, United States of America; 4 Division of Viral Hepatitis, National Center for HIV, Viral Hepatitis, STD and TB Prevention, Centers for Disease Control and Prevention, Atlanta, Georgia, United States of America; Kaohsiung Medical University, TAIWAN

## Abstract

Hepatitis A virus can cause severe and prolonged illness in persons with HIV (PWH). In July 2020, the Advisory Committee on Immunization Practices (ACIP) expanded its recommendation for hepatitis A vaccination to include all PWH aged ≥1 year. We used a decision analytic model to estimate the value of vaccinating a cohort of adult PWH aged ≥20 years with diagnosed HIV in the United States using a limited societal perspective. The model compared 3 scenarios over an analytic horizon of 1 year: no vaccination, current vaccine coverage, and full vaccination. We incorporated the direct medical costs and nonmedical costs (i.e., public health costs and productivity loss). We estimated the total number of infections averted, cost to vaccinate, and incremental cost per case averted. Full implementation of the ACIP recommendation resulted in 775 to 812 fewer adult cases of hepatitis A in 1 year compared with the observed vaccination coverage. The incremental cost-effectiveness ratio for the full vaccination scenario was $48,000 for the 2-dose single-antigen hepatitis A vaccine and $130,000 for the 3-dose combination hepatitis A and hepatitis B vaccine per case averted, compared with the observed vaccination scenario. Depending on type of vaccine, full hepatitis A vaccination of PWH could lead to ≥80% reduction in the number of cases and $48,000 to $130,000 in additional cost per case averted. Data on hepatitis A health outcomes and costs specific to PWH are needed to better understand the longer-term costs and benefits of the 2020 ACIP recommendation.

## Introduction

Unvaccinated persons with HIV are susceptible to hepatitis A virus (HAV) infection and if infected, are at increased risk for higher HAV viral load and prolonged viremia [1,2]; persons with HIV infection (PWH) who have underlying liver disease are at increased risk for severe hepatitis A [3]. In July 2020, the Advisory Committee on Immunization Practices (ACIP) expanded its recommendation for hepatitis A vaccination to include all PWH aged ≥1 year [4].

The benefits and potential cost-effectiveness of vaccinating children and adults at risk for HAV infection or for severe disease from HAV infection are well established. While catch-up vaccination among unvaccinated children and adolescents has been found to be cost-saving compared with routine vaccination [5], other studies suggest that hepatitis A vaccination is potentially cost-effective under narrow criteria (for example, universal compared to regional vaccination, vaccination at high incidence levels, and vaccination of populations at risk for severe disease). Although the ACIP specifically addresses vaccination of PWH as a group at risk for severe disease, there are no published studies on the cost-effectiveness of hepatitis A vaccination among PWH in the United States. We modeled the potential cost-effectiveness of hepatitis A vaccination among adult PWH in the United States using the 2-dose single-antigen hepatitis A vaccine and the 3-dose combination hepatitis A and B vaccine. We estimated the total cost to vaccinate a cohort of adult PWH in 2019 and the number and associated cost of infections that would be averted in this population over a 1-year period.

## Methods

We developed a decision analytic model to assess the value of hepatitis A vaccination among adult PWH. We compared 3 scenarios: 1) no vaccination, 2) observed vaccination coverage, and 3) full vaccination or 100% vaccination coverage (S1 and S2 Figs). We determined the total number of infections averted in 1 year, cost to vaccinate a cohort of PWH, and incremental cost-effectiveness of the vaccination recommendation. We calculated the incremental cost-effectiveness ratio (ICER) as the difference between total costs divided by the difference in outcomes (defined as hepatitis A cases) between scenarios.

We adopted a limited societal perspective in estimating costs, which incorporated the direct medical costs and nonmedical costs (i.e., public health costs and productivity loss due to illness). The direct medical costs included outpatient and inpatient costs of clinical management of HAV infection, vaccine and vaccine administration costs, and the cost of postvaccination serologic testing for HAV immunity [6–11]. We included the full cost of the 3-dose hepatitis A and hepatitis B combination vaccine and the 2-dose hepatitis A single-antigen vaccine. All direct medical costs were derived from the general population from published literature because costs specific to PWH were not available. For public health costs, we relied on published estimates of resources expended to conduct case investigations for a case of HAV infection in an outbreak setting [12]. We excluded the cost of adverse events due to vaccination and the cost of disease sequelae among PWH because of data availability. We also excluded out-of-pocket costs for PWH seeking care assuming some or all of vaccine doses and serological testing received occurred as part of other routine medical care. The model estimated intermediate health outcomes and costs over an analytic horizon of 1 year because of limitations in cost and long-term outcomes data for HAV infections among PWH. All cost estimates were adjusted to 2019 US dollars using the Bureau of Economic Analysis price indexes for personal consumption expenditures for health care [13].

Productivity loss was calculated using household income information for PWH, adjusted for the number employed among the cohort, and multiplied by the total number of hours of work missed due to illness (Table 1) [12,14]. We incorporated both public and private price for the hepatitis A vaccine using the Centers for Disease Control and Prevention’s (CDC) vaccine price list [6]. The vaccine price was calculated by weighting the proportion of PWH who reported private health insurance in the CDC Medical Monitoring Project (MMP) multiplied by the private price plus the proportion of PWH in any other payer type (i.e., Ryan White HIV/AIDS program, Medicaid, Medicare, Tricare, Veterans Administration, or Other) multiplied by the public price [15].

**Table 1:** Model inputs.

Model Inputs	Base	Lower	Upper	Source
**Costs (2019 US dollars)**				
Vaccine (cost per dose [$], 2-dose single-antigen vaccine; 3-dose combination vaccine)*	43; 73	28; 57	68; 101	[6,14]
Vaccine administration (cost per dose administered [$])^†^	16	12	18	[9,10]
Hepatitis A antibody testing ($)^§^	28	13	54	[7,8]
Inpatient^Δ^	17,024	15,787	18,261	[11]
Outpatient^ΔΔ^	1,188	850	1,424	[10]
Total inpatient and outpatient ($)	18,212	16,637	19,685	[10,11]
Public health case investigation (cost per case[$])**	386	193	772	[12]
Household income^#^ ($)	18,500	17,000	19,999	[14]
Work loss per patient (days)	12	0	42	[12]
Labor force participation (%)	40.6	38.5	42.6	[14]
**Hepatitis A and vaccination rates among persons with HIV (PWH)**				
Number of persons aged ≥20 years with diagnosed HIV^††^	1,038,068	Not included	Not included	[19]
Hepatitis A annual incidence (%)^§§^	0.25	0.15	0.3	[14]
*Single-antigen vaccine (hepatitis A vaccine)*				
Seroconversion rate after 1 dose (%)	72	51	99	[18]
Seroconversion rate after 2 doses (%)	89	83	100	[17,18]
*Combination vaccine (hepatitis A and hepatitis B vaccine)*				
Seroconversion rate after 1 dose (%)^¶¶^	93.8	Not included	Not included	[16]
Seroconversion rate after 2 doses (%)	98.8	Not included	Not included	[16]
Seroconversion rate after 3 doses (%)	99.9	97.9	100	[16]
Fully vaccinated (%)***	22.1	19.5	24.7	[14]
Susceptible to infection (%)^†††^	46.8	43.9	49.7	[14]
Received 1 dose [%] single-antigen; combination vaccine	35; 30.1	Not included	Not included	[14]
Received 2 doses [%] combination vaccine	4.9	Not included	Not included	[14]
Immune from previous infection (%)^§§§^	31.1	28.6	33.6	[14]

* The base vaccine cost per dose (2019 USD) was weighted using patient insurance status (i.e., 36% of PWH are eligible for vaccine under a private insurance price) [15]. Using the Centers for Disease Control and Prevention’s (CDC) January 2019 adult vaccine price list, the weighted price for a 2-dose series of single-antigen vaccine was calculated as the private price cost per dose multiplied by the share of PWH under private insurance plus the public price cost per dose multiplied by the share of PWH under ‘any other insurance type’ (proxy for government or public insurance). The lower and upper bound vaccine cost per dose reflects unweighted prices from CDC’s January 2019 adult vaccine price list.

^†^The base vaccine administration cost (2019 USD) was weighted using insurance status [15]. The 2019 Centers for Medicare and Medicaid Services (CMS) reimbursement for immunization administration (HCPCS code 90471) represented the public price, and the vaccine administration cost from published literature represented the private price.

^§^ We approximated the cost of post-vaccination serologic testing (2019 USD) using the CMS cost for IgM antibody testing. The base cost for immunoglobulin testing (IgM) was weighted using insurance status [15]. The 2019 CMS reimbursement for clinical diagnostic laboratory fee for hepatitis A IgM antibody (HCPCS code 86709) represented the public price, and the vaccine administration cost from Find Lab Test was used as the private price.

^¶^Adverse events following vaccination include injection site reactions and mild systemic reactions [4]. Therefore, we did not include the cost of adverse events in the model.

^Δ^Inpatient cost (originally 2017 USD) was adjusted for inflation using the Bureau of Economic Analysis (BEA) index for personal consumption expenditures (PCE) table 2.4.4U line 180 ‘Hospital and nursing home services’ [13].

^ΔΔ^Outpatient cost (originally 2013 USD) was adjusted for inflation using the BEA index for PCE table 2.4.4U line 171 ‘Outpatient services’ [13].

**The public health investigation cost (originally 1999 USD) upper and lower bounds were assumed to be half and double the base value, which was derived from published literature. This cost was adjusted for inflation using the BEA index for PCE table 2.4.4U line 170 ‘health care’.

^#^Household income (2019 USD) were derived from MMP.

^††^ AtlasPlus reports the number of people with diagnosed HIV aged ≥13 years. The number aged ≥20 years was calculated by subtracting the share in the 13–19 age group (i.e., HIV prevalence for aged ≥13 years minus the HIV prevalence among adolescents [aged 13–19 years]) [20] from AtlasPlus [19].

^§§^The annual incidence for hepatitis A was based on an estimated 2-year prevalence of diagnosed hepatitis A among people with diagnosed HIV receiving HIV care.

^¶¶^Estimates of seroconversion for the combination vaccine are among the general adult population.

***Represents all people with diagnosed HIV who were fully vaccinated. The lower and upper bound represent the 95% confidence interval (CI) from the sampled population in MMP.

^†††^Susceptible to infection/not immune (not fully vaccinated, HAVAb negative or missing, and no documentation that reason for not vaccinating was previous infection); assumes vaccine is 100% effective. The lower and upper bound represent the 95% CI from the sampled population in MMP.

^§§§^Immune from previous infection (documentation that previous infection was reason for not vaccinating or HAVAb positive and not fully vaccinated). The lower and upper bound represent the 95% CI from the sampled population in MMP.

Three scenarios were compared in the model: no vaccination, current vaccine coverage, and full vaccination. For the no vaccination scenario (0%), we estimated the number of HAV infections expected over the duration of 1-year in the absence of vaccination. In the observed vaccination coverage scenario, we estimated the status quo vaccination coverage of hepatitis A vaccination (22.1% of PWH fully vaccinated) before the ACIP recommendation in July 2020 from unpublished MMP data [14]. The MMP is a CDC surveillance system designed to produce nationally representative estimates of the behavioral and clinical characteristics of adults aged ≥18 years with diagnosed HIV in the United States, of which a subset receive HIV medical care [14]. The MMP surveillance system has been described extensively in the published literature. Lastly, we assumed 100% coverage in the full vaccination scenario.

We estimated the percentage of PWH with no hepatitis A vaccination, partial vaccination (≥1 dose of single-antigen or combination vaccine), and full vaccination (≥2 doses of single-antigen vaccine or ≥3 doses of combination vaccine) using 2009–2012 unpublished MMP data, which represented the most recent timeframe with available vaccination coverage data. This estimate was generated separately for the single-antigen and combination vaccines and excluded PWH who received both vaccine types. For the full vaccination scenario, we assumed 100% vaccine acceptance (i.e., all PWH without prior history of vaccination or immunity from prior infection would receive 2 doses of the single-antigen vaccine or 3 doses of the combination vaccine).

The annual incidence of diagnosed HAV infection among PWH was derived from unpublished MMP data based on a 2-year observation period of PWH at their usual place of HIV care and excludes undiagnosed HAV infection [14]. The 2-year prevalence was divided by 2 to approximate an annual incidence rate. Furthermore, we incorporated the probability of prior immunity among PWH from MMP for each scenario. We obtained seroprotection rates for each dose and type of vaccine received [16–18]. Seroprotection rates among PWH by vaccine dose were used when available (i.e., single-antigen vaccine); seroprotection rates by dose for the general population were used when not available for PWH (i.e., combination vaccine).

The model was applied to a cohort of adults with diagnosed HIV in 2019 in the United States using diagnosed HIV prevalence data in AtlasPlus [19]. There were 1,043,284 persons aged ≥13 years with diagnosed HIV in the United States in 2019. We excluded diagnoses among persons aged 13–19 using 2016 surveillance data on HIV prevalence among adolescents (0.5% of the PWH cohort in 2019) [20]. The final cohort size represented adults aged ≥20 years (N = 1,038,068).

The model was built using TreeAge Pro 2021, R1 Healthcare (TreeAge Software, Williamstown, MA). We conducted a sensitivity analysis in TreeAge Pro to determine the inputs of highest influence on the ICER using lower and upper bound estimates of input parameter ranges when available from the literature. When input ranges were not available, specifically for costs, we used half and double the base values to represent the lower and upper bound estimates (Table 1). We also conducted threshold analysis on selected inputs with the greatest influence on the ICER.

## Results

Table 2 presents results of hepatitis A vaccination for a cohort of 1,038,068 adults with diagnosed HIV in 2019 by vaccine type. Among PWH, no vaccination resulted in 1,788 cases of HAV infections in 1 year. Compared with no vaccination, observed vaccination represented a 46% (single-antigen vaccine) to 55% (combination vaccine) reduction in hepatitis A cases among PWH. Full vaccination represented 80% (single-antigen vaccine) to 99% (combination vaccine) reduction in hepatitis A cases among PWH compared with observed vaccination.

**Table 2:** Costs and effectiveness of hepatitis A vaccination among persons with HIV (PWH), United States, 2019, N = 1,038,068.

	Costs Per Scenario^†^	Number Vaccinated^§^	Incremental Cost^¶^	Hepatitis A Cases**	Hepatitis A Cases Averted^††^	Incremental Cost-Effectiveness Ratio^§§^
Single-antigen vaccine*						
No vaccination	$33,925,079	—	—	1,788	—	—
Observed vaccination	$66,720,854	402,770	$32,795,775	972	816	$40,141
Full vaccination	$104,479,968	726,648	$37,759,114	197	775	$48,721
Combination vaccine						
No vaccination	$33,925,079	—	—	1,788	—	—
Observed vaccination	$105,122,316	402,770	$71,197,239	813	975	$73,022
Full vaccination	$210,958,843	726,648	$105,836,525	1	812	$130,340

*Two-dose series using single-antigen vaccine or 3-dose series using combination vaccine.

†Cost per scenario. Each cell represents the total cost for each scenario. The total cost for each scenario is equal to the sum of 4 components: (1) cost of vaccine, vaccine administration, and postvaccination serologic testing; (2) cost of supportive care/management for hepatitis A; (3) cost of public health investigation for hepatitis A; and (4) cost of productivity loss.

^§^Number of PWH vaccinated. The first cell (no vaccination) represents a null value or 0 doses; the second cell (observed vaccination) represents 402,770 (252,790 fully vaccinated and 149,980 partially vaccinated); while the last cell (full vaccination) represents 726,648 who received 2 or 3 doses of hepatitis A or combination hepatitis A and hepatitis B vaccine, respectively.

^¶^Incremental cost. Each cell represents the difference in costs between 2 scenarios. Scenarios were ranked by order of effectiveness (i.e., by Column 6, with no vaccination being the least effective). In the top panel of Table 2, the incremental cost of the observed vaccination scenario is $66,720,854– $33,925,079 = $32,795,775. The incremental cost of the full vaccination scenario is $104,479,968 –$66,720,854 = $37,759,114. In the bottom panel, the incremental cost of the observed vaccination scenario is $105,122,316 –$33,925,079 = $71,197,239. The incremental cost of the full vaccination scenario is $210,958,843 –$105,122,318 = $105,836,525.

**Hepatitis A cases. This represents the total number of hepatitis A cases estimated under each scenario for a cohort of PWH in 2019 (N = 1,038,068). In the top panel representing the 2-dose hepatitis A series, there were 1,788 hepatitis A cases in the no vaccination scenario, 972 cases in the observed vaccination scenario, and 197 cases in the full vaccination scenario. In the bottom panel representing the combination vaccine, there were 1,788 hepatitis A cases in the no vaccination scenario, 813 cases in the observed vaccination scenario, and 1 case in the full vaccination scenario.

^**††**^This represents the incremental difference in cases between 2 scenarios. In the top panel, the incremental cases or cases averted for the observed vaccination scenario is the total number of cases under no vaccination (in Column 5) minus the total number of cases under observed vaccination (in Column 5) or 1,788–972 = 816, while the incremental cases or cases averted for the full vaccination scenario is total number of cases under the observed vaccination scenario (second cell in Column 5) minus the total number of cases under the full vaccination scenario or 972–197 = 775 (third cell in Column 5). In the bottom panel, the incremental cases or cases averted for the observed vaccination scenario is the total number of cases under no vaccination (in Column 5) minus the total number of cases under observed vaccination (in Column 5) or 1,788–813 = 975, while the incremental cases or cases averted for the full vaccination scenario is total number of cases under the observed vaccination scenario (bottom panel, second cell in Column 5) minus the total number of cases under the full vaccination scenario or 813–1 = 812 (bottom panel, third cell in Column 5).

^**§§**^The cells in this column represent the incremental cost-effectiveness ratio (ICER) or the ratio of incremental costs (Column 4) to incremental cases (Column 6). The ICER represents the incremental cost per case of hepatitis A averted under each scenario. In the top panel, the ICER for the observed vaccination scenario is $32,795,775 / 816 cases = $40,141, while the ICER for the full vaccination is $37,759,114 / 775 cases = $48,721. In the bottom panel, the ICER for the observed vaccination scenario is $71,197,239 / 975 cases = $73,022, while the ICER for the full vaccination is $105,836,525 / 812 cases = $130,340.

There was a marked difference in costs between full vaccination and observed vaccination. The incremental cost of $37 million (single-antigen vaccine) to $105 million (combination vaccine) between full vaccination and observed vaccination reflects the magnitude of scale-up needed to vaccinate all PWH with no prior history of HAV infection or partially vaccinated. The higher ICER for the combination vaccine under the full vaccination scenario reflects the additional three hundred thousand PWH needing one more dose of vaccine to complete the three dose vaccine series, and the higher vaccine price of the combination vaccine. Overall, full vaccination resulted in a cost of $144 per PWH vaccinated with the single-antigen vaccine and $290 per PWH vaccinated with the combination vaccine. Comparing the incremental cost-effectiveness of full vaccination to observed vaccination resulted in an incremental cost per case averted of $48,721 (single-antigen vaccine) to $130,340 (combination vaccine).

### Sensitivity analysis

**Fig 1:**
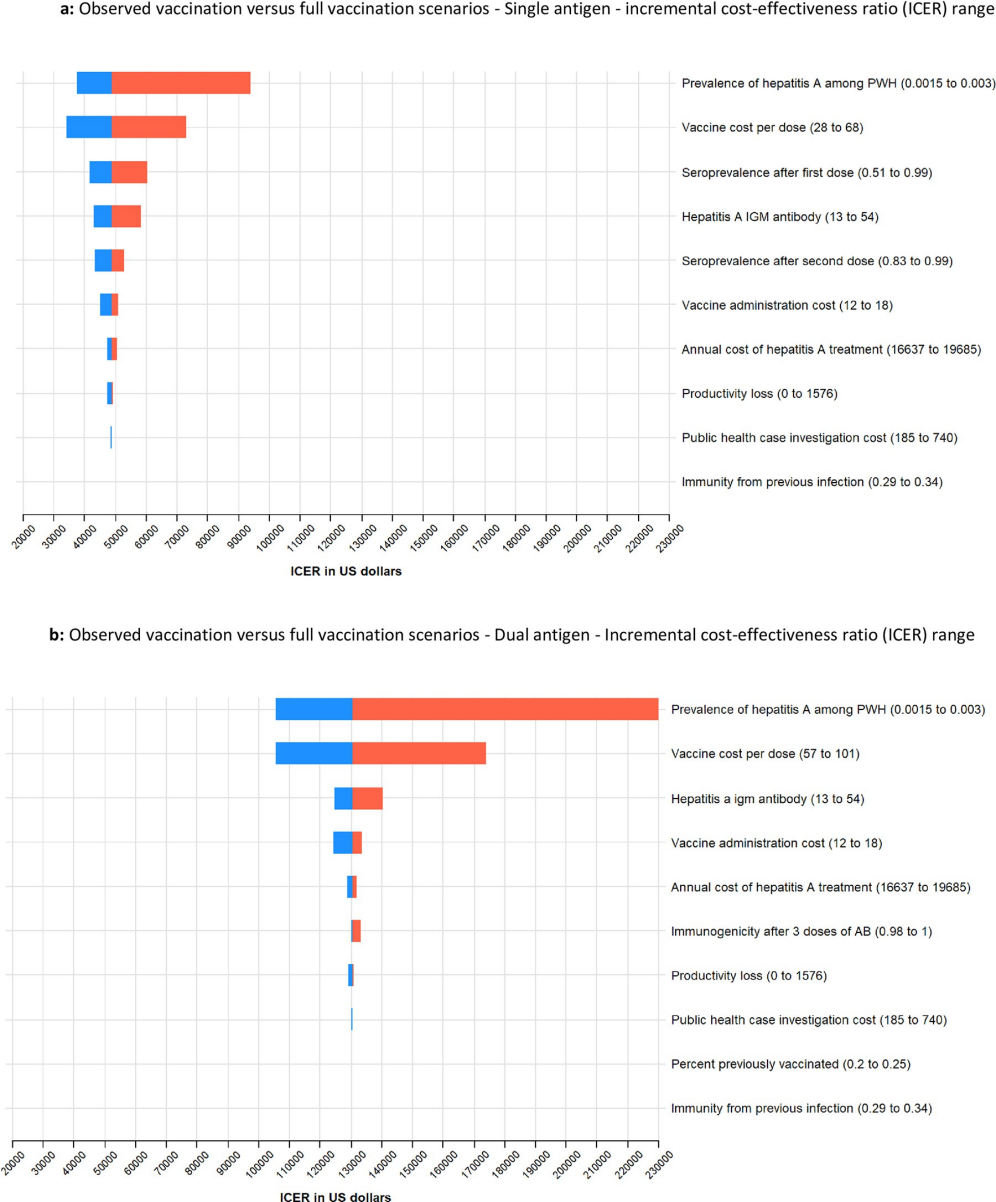
Cost-Effectiveness of hepatitis A vaccination among adults with diagnosed HIV, United States. a. Observed vaccination versus full vaccination scenarios—Single antigen—incremental cost-effectiveness ratio (ICER) range. This figure shows a Tornado plot of the modeled inputs for the single antigen vaccine and range of ICERs for each modeled input. b. Observed vaccination versus full vaccination scenarios—Single antigen—incremental cost-effectiveness ratio (ICER) range. This figure shows a Tornado plot of the modeled inputs for the dual antigen vaccine and range of ICERs for each modeled input.

**Fig 2:**
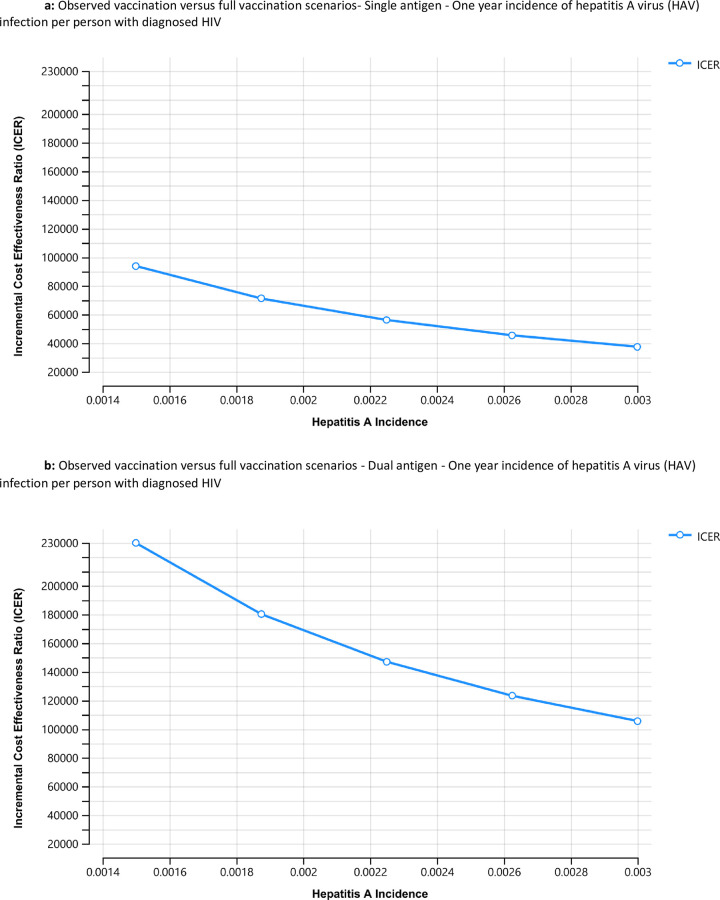
a. Observed vaccination versus full vaccination scenarios—Single antigen—One year incidence of hepatitis A virus (HAV) infection per person with diagnosed HIV. This figure shows sensitivity analysis based on incidence of hepatitis A virus infection comparing observed vaccination with full vaccination for the dual antigen vaccine. The Y axis represents the incremental cost effectiveness ratio (ICER). The X axis represents the range of incidence of hepatitis A between 0.0014 and 0.003. b. Observed vaccination versus full vaccination scenarios—Dual antigen—One year incidence of hepatitis A virus (HAV) infection per person with diagnosed HIV. This figure shows sensitivity analysis based on incidence of hepatitis A virus infection comparing observed vaccination with full vaccination for the dual antigen vaccine. The Y axis represents the incremental cost effectiveness ratio (ICER). The X axis represents the range of incidence of hepatitis A between 0.0014 and 0.003.

**Fig 3:**
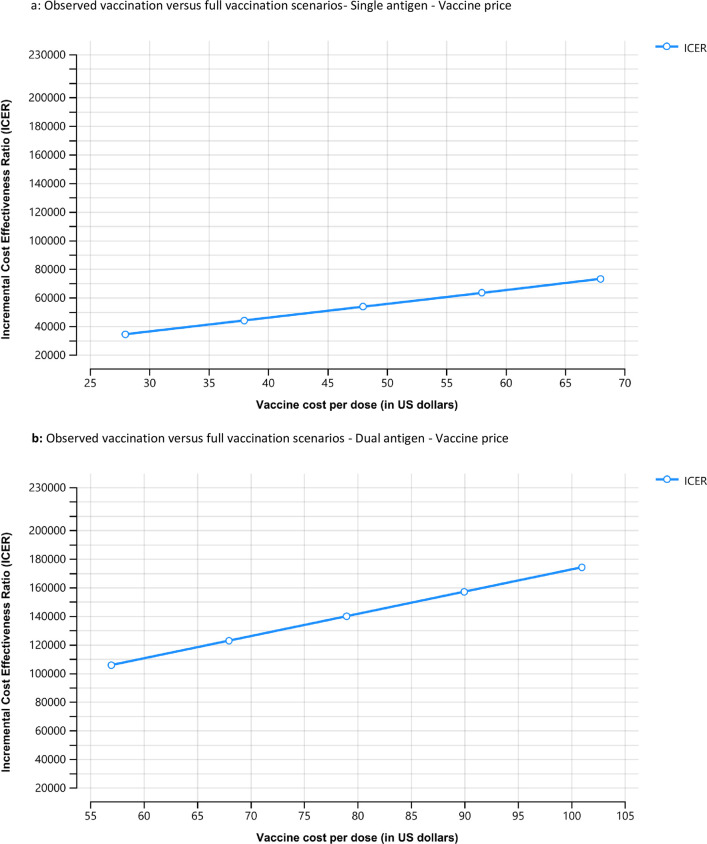
a. Observed vaccination versus full vaccination scenarios- Single antigen—Vaccine price. This figure shows sensitivity analysis based on vaccine cost per dose comparing observed vaccination with full vaccination for the single antigen vaccine. The Y axis represents the incremental cost effectiveness ratio (ICER). The X axis represents the range of vaccine cost per dose in US dollars between $25 and $70. b. Observed vaccination versus full vaccination scenarios- Dual antigen—Vaccine price. This figure shows sensitivity analysis based on vaccine cost per dose comparing observed vaccination with full vaccination for the dual antigen vaccine. The Y axis represents the incremental cost effectiveness ratio (ICER). The X axis represents the range of vaccine cost per dose in US dollars between $55 and $105.

The results of the sensitivity analysis, using a Tornado plot and one-way diagrams for the single-antigen vaccine, are presented in Figs 1A, 2A and 3A and show the high and low values of the data inputs and the resulting ICER. The incidence of HAV infection and the price of vaccine per dose produced the widest range of uncertainty in the ICER results. The resulting ICERs for full vaccination increased with declining levels of hepatitis A incidence, suggesting that observed vaccination could become superior to full vaccination at low levels of hepatitis A incidence (Fig 2A). Conversely, the resulting ICERs for full vaccination increase with the vaccine price suggesting less favorability of full vaccination as the vaccine price approaches the upper bound (Fig 3A). Similar sensitivity analysis conducted for the combination vaccine are presented in Figs 1B, 2B and 3B.

## Discussion

This study presents findings on the cost-effectiveness of full hepatitis A vaccination among PWH. When compared with the observed vaccination coverage prior to the 2020 ACIP recommendation, full vaccination of adult PWH could lead to ≥80% reduction in the number of hepatitis A cases at an incremental cost per case averted of $48,000 (single-antigen vaccine) to $130,000 (combination vaccine).

The benefits and costs of vaccinating children and adults at risk for severe disease from HAV infection, such as persons with chronic liver disease (e.g., hepatitis C), have previously been documented [21–23]. Studies have considered various factors influencing cost-effectiveness of hepatitis A vaccination, including hepatitis A incidence, age, and severity of hepatitis C disease. Arguedas et al [21]. compared no hepatitis A vaccination to targeted hepatitis A vaccination among adults with late-stage hepatitis C and estimated an ICER of $51,000 per quality-adjusted life-year. Myers et al [23]. evaluated 3 strategies for hepatitis A vaccination among adults with hepatitis C (vaccinate no patients, vaccinate only patients testing HAV negative, and vaccinate all patients) and estimated an ICER of $20.1 million per life-year saved. Jacobs et al [22]. considered an age-based strategy (i.e., vaccination at ages 30, 45, and 60 years) and found the lowest ICER in those aged 30 years.

Our findings are subject to several limitations. We did not study sex and age, though analysis by sex or age is not expected to substantially change our findings. HAV disease severity increases with age and immune response is less robust among older age groups (>40 years) [24]. All licensed hepatitis A vaccines are highly immunogenic in adults aged >18 years when administered according to the recommended schedules and limited data are available regarding the timing needed to develop protective antibodies. However, studies have shown similar response among age groups at 30 days post vaccination, with lower response in older age groups at 15 days post vaccination [4]. Response to vaccine by sex has not been widely studied but thought to be similar among groups. While two studies have shown female sex to be associated with better vaccine response [25,26], others found male sex to be associated with better response [27,28].

The vaccination coverage from MMP only reflects PWH who know their HIV status and are in care. Undiagnosed HIV or PWH who do not know their status or those who received a diagnosis but were lost to follow-up were not included; these persons not in care are potentially the most vulnerable to HAV infection. Our study assumes a constant scale of production costs needed to attain 100% vaccination coverage, while vaccinating every PWH may require a higher level of resource intensity not considered in the model. With no data on health outcomes for HAV infection specific to PWH, we chose a simple decision tree model to estimate costs and effectiveness rather than a dynamic model that would require data not available to appropriately develop and interpret the findings. We limit the analytic horizon of the model to 1-year and could not assess the full benefits of hepatitis A vaccination (including disease incidence beyond the analytic horizon, prevention of fulminant hepatitis A, liver transplantation, and years of life lost due to disease).

Similarly, there is a lack of data on the cost of supportive medical care for HAV infections among PWH, including outpatient management of severe infection and hospitalization. Therefore, we infer these costs from the general population, potentially underestimating the cost of HAV infection management for PWH and the cost-effectiveness of full vaccination of PWH. We conservatively estimated the cost-effectiveness of full vaccination with the combination vaccine by including the entire cost of the vaccine in the model. In practice, a portion of this cost would be attributed to hepatitis B vaccination. While the full cost of the combination vaccine was included in our model, it is worth noting that we did not include the benefits of hepatitis B vaccine since it falls outside the scope of our model. Excluding the benefits of hepatitis B vaccination underestimates the full benefit of the combination vaccine; however, the full cost of the combination vaccine is incurred when administered in a real-world scenario regardless of if protection is desired against hepatitis A, hepatitis B, or both. As such, our results should not be interpreted as a head-to-head comparison of vaccine types, but instead as a range of cost-effectiveness estimates specific to the modeled hepatitis A vaccination scenarios.

Our study perspective excluded aspects of patient costs and benefits for which data were not readily available to quantify (such as caregiving costs and costs related to intangible pain/suffering) or were outside the 1-year analytic horizon (such as life years gained or lost). Inclusion of additional patient costs and benefits of averting hepatitis A would contribute to decreasing the ICER in favor of full vaccination for PWH.

Published data on combination hepatitis A and hepatitis B vaccine seroconversion among PWH are limited, and only 3 published studies were found that provide separate seroconversion information by dose for the combination vaccine schedule. Fritzsche et al [26]. provided a seroconversion rate for the combination vaccine but only for the third dose. Jimenez et al [29]. does not provide a breakdown of seroconversion between the first and second doses of the combination vaccine. Lastly, Mena et al [30]. offers seroconversion rates for the accelerated vaccination schedule, which is not the focus of our model. We used the Twinrix (GlaxoSmithKline Biologicals, Rixensart, Belgium) packet insert estimates of seroconversion among the general population because it offers separate seroconversion rates for each dose of the vaccination schedule. The reliance on general population seroconversion rates in our model is likely a substantial overestimate of the number of PWH who seroconvert with a combination hepatitis A and hepatitis B vaccine.

Full hepatitis A vaccination among adult PWH, as recommended by ACIP, would produce large reductions in the number of hepatitis A cases. Depending on type of vaccine, full hepatitis A vaccination may generate $48,000 to $130,000 in additional cost per case averted compared with the status quo for hepatitis A vaccination. Data on hepatitis A health outcomes and costs specific to PWH are needed to better understand the longer-term costs and health benefits of the 2020 ACIP recommendation for PWH.

## Supporting information

S1 Fig Model structure for the single-antigen vaccine. This figure shows the model structure or schematic developed in TreeAge Pro for the single antigen vaccine.
(PDF)

S2 Fig Model structure for the combination vaccine. This figure shows the model structure or schematic developed in TreeAge pro for the dual antigen vaccine.
(PDF)

## References

[1] Ida S, Tachikawa N, Nakajima A, Daikoku M, Yano M, Kikuchi Y, et al. Influence of human immunodeficiency virus type 1 infection on acute hepatitis A virus infection. Clin Infect Dis. 2002;34(3):379–85. doi: 10.1086/338152 11774086

[2] Lin KY, Chen GJ, Lee YL, Huang YC, Cheng A, Sun HY, et al. Hepatitis A virus infection and hepatitis A vaccination in human immunodeficiency virus-positive patients: A review. World J Gastroenterol. 2017;23(20):3589–606. doi: 10.3748/wjg.v23.i20.3589 28611512 PMC5449416

[3] Laurence JC. Hepatitis A and B immunizations of individuals infected with human immunodeficiency virus. Am J Med. 2005;118 Suppl 10A:75S–83S. doi: 10.1016/j.amjmed.2005.07.024 16271546

[4] Nelson NP, Weng MK, Hofmeister MG, Moore KL, Doshani M, Kamili S, et al. Prevention of hepatitis A virus infection in the United States: Recommendations of the advisory committee on immunization practices, 2020. MMWR Recomm Rep. 2020;69(5):1–38. doi: 10.15585/mmwr.rr6905a1 32614811 PMC8631741

[5] Elbasha EH, Choi Y, Daniels V, Goveia MG. Cost-effectiveness of routine catch-up hepatitis a vaccination in the United States: Dynamic transmission modeling study. Vaccine. 2021;39(42):6315–21. doi: 10.1016/j.vaccine.2021.08.087 34538694

[6] Archived CDC vaccine price list as of January 2, 2019 [Available from: https://www.cdc.gov/vaccines/programs/vfc/awardees/vaccine-management/price-list/2019/2019-01-02.html.

[7] Centers for Medicare and Medicaid Services. Clinical laboratory fee schedules [Internet]. [cited September 23, 2021]. Available from: https://www.cms.gov/Medicare/Medicare-Fee-for-Service-Payment/ClinicalLabFeeSched/Clinical-Laboratory-Fee-Schedule-Files.

[8] Find lab test. Hepatitis A test cost [updated September 23, 2021. Available from: https://www.findlabtest.com/lab-test/infectious-disease-testing/hepatitis-a-test-cost-quest-508?verbose=0.

[9] Centers for Medicare and Medicaid Services. Selected Procedures CMS-1693-F. 26110197

[10] Dhankhar P, Nwankwo C, Pillsbury M, Lauschke A, Goveia MG, Acosta CJ, et al. Public Health Impact and Cost-Effectiveness of Hepatitis A Vaccination in the United States: A Disease Transmission Dynamic Modeling Approach. Value Health. 2015;18(4):358–67. doi: 10.1016/j.jval.2015.02.004 26091589

[11] Hofmeister MG, Yin S, Aslam MV, Teshale EH, Spradling PR. Hepatitis A hospitalization costs, United States, 2017. Emerg Infect Dis. 2020;26(5):1040–1. doi: 10.3201/eid2605.191224 32310068 PMC7181923

[12] Sansom SL, Cotter SM, Smith F, Koch E, de Fijter S, Long T, et al. Costs of a hepatitis A outbreak affecting homosexual men: Franklin County, Ohio, 1999. Am J Prev Med. 2003;25(4):343–6. doi: 10.1016/s0749-3797(03)00209-5 14580638

[13] U.S. Bureau of Economic Analysis. Table 2.4.4U. Price Indexes for Personal Consumption Expenditures by Type of Product, [Available from: https://apps.bea.gov/iTable/?1921=underlying&isuri=1&reqid=19&step=2#eyJhcHBpZCI6MTksInN0ZXBzIjpbMSwyLDMsM10sImRhdGEiOltbIkNhdGVnb3JpZXMiLCJTdXJ2ZXkiXSxbIk5JUEFfVGFibGVfTGlzdCIsIjIwMTYiXSxbIkZpcnN0X1llYXIiLCIxOTc5Il0sWyJMYXN0X1llYXIiLCIyMDE5Il0sWyJTY2FsZSIsIjAiXSxbIlNlcmllcyIsIkEiXV19.

[14] CDC, Unpublished. Medical Monitoring Project (MMP) [Available from: https://www.cdc.gov/hiv/statistics/systems/mmp/index.html.

[15] Centers for Disease Control and Prevention. HIV surveillance special report 25. Behavioral and clinical characteristics of persons with diagnosed HIV infection—Medical Monitoring Project, United States, 2018 Cycle (June 2018–May 2019). May 2020.

[16] U.S. Food and Drug Administration. Twinrix package insert. https://www.fda.gov/vaccines-blood-biologics/vaccines/twinrix.

[17] Crum-Cianflone NF, Wilkins K, Lee AW, Grosso A, Landrum ML, Weintrob A, et al. Long-term durability of immune responses after hepatitis A vaccination among HIV-infected adults. J Infect Dis. 2011;203(12):1815–23. doi: 10.1093/infdis/jir180 21606540 PMC3100512

[18] Wallace MR, Brandt CJ, Earhart KC, Kuter BJ, Grosso AD, Lakkis H, et al. Safety and immunogenicity of an inactivated hepatitis A vaccine among HIV-infected subjects. Clin Infect Dis. 2004;39(8):1207–13. doi: 10.1086/424666 15486846

[19] Centers for Disease Control and Prevention. NCHHSTP AtlasPlus [Available from: https://www.cdc.gov/nchhstp/atlas/index.htm.

[20] Centers for Disease Control and Prevention. Diagnoses of HIV infection among adolescents and young adults in the United States and 6 dependent areas, 2012–2017. HIV surveillance supplemental report 2019;24 (No. 5).

[21] Arguedas MR, Heudebert GR, Fallon MB, Stinnett AA. The cost-effectiveness of hepatitis A vaccination in patients with chronic hepatitis C viral infection in the United States. Am J Gastroenterol. 2002;97(3):721–8. doi: 10.1111/j.1572-0241.2002.05554.x 11922569

[22] Jacobs RJ, Koff RS, Meyerhoff AS. The cost-effectiveness of vaccinating chronic hepatitis C patients against hepatitis A. Am J Gastroenterol. 2002;97(2):427–34. doi: 10.1111/j.1572-0241.2002.05481.x 11866283

[23] Myers RP, Gregor JC, Marotta PJ. The cost-effectiveness of hepatitis A vaccination in patients with chronic hepatitis C. Hepatology. 2000;31(4):834–9. doi: 10.1053/he.2000.5719 10733536

[24] Centers for Disease Control and Prevention. Viral Hepatitis Surveillance Report–United States, 2020. https://www.cdc.gov/hepatitis/statistics/2020surveillance/index.htm. Published September 2022.

[25] Weissman S, Feucht C, Moore BA. Response to hepatitis A vaccine in HIV-positive patients. J Viral Hepat. 2006;13(2):81–6. doi: 10.1111/j.1365-2893.2005.00658.x 16436125

[26] Fritzsche C, Bergmann L, Loebermann M, Glass A, Reisinger EC. Immune response to hepatitis A vaccine in patients with HIV. Vaccine. 2019;37(16):2278–83. doi: 10.1016/j.vaccine.2019.02.064 30890384

[27] Armstrong KE, Bush HM, Collins JD, Feola DJ, Caldwell GC, Thornton AC. Role of CD4 count in immunity development after hepatitis A and B vaccination among HIV-infected patients: Kentucky, 2002–2007. J Int Assoc Physicians AIDS Care (Chic). 2010;9(3):179–86. doi: 10.1177/1545109710368721 20530473

[28] Overton ET, Nurutdinova D, Sungkanuparph S, Seyfried W, Groger RK, Powderly WG. Predictors of immunity after hepatitis A vaccination in HIV-infected persons. J Viral Hepat. 2007;14(3):189–93. doi: 10.1111/j.1365-2893.2006.00822.x 17305885

[29] Jimenez HR, Hallit RR, Debari VA, Slim J. Hepatitis A vaccine response in HIV-infected patients: are TWINRIX and HAVRIX interchangeable? Vaccine. 2013;31(9):1328–33. doi: 10.1016/j.vaccine.2012.12.045 23277097

[30] Mena G, Garcia-Basteiro AL, Llupia A, Diez C, Costa J, Gatell JM, et al. Factors associated with the immune response to hepatitis A vaccination in HIV-infected patients in the era of highly active antiretroviral therapy. Vaccine. 2013;31(36):3668–74. doi: 10.1016/j.vaccine.2013.06.012 23777950

