# Supplementary material for: Cost-effectiveness of expanded hepatitis A vaccination among adults with diagnosed HIV, United States
Source: PLoS One. 2023 Mar 17;18(3):e0282972. doi: 10.1371/journal.pone.0282972 (PMC10022807; doi:10.1371/journal.pone.0282972)
Supplement: S1 Fig — This figure shows the model structure or schematic developed in TreeAge Pro for the single antigen vaccine. (PDF) [file pone.0282972.s001.pdf]

Supplemental Figures: Cost-Effectiveness of Hepatitis A Vaccination Among Adults with Diagnosed HIV, United States

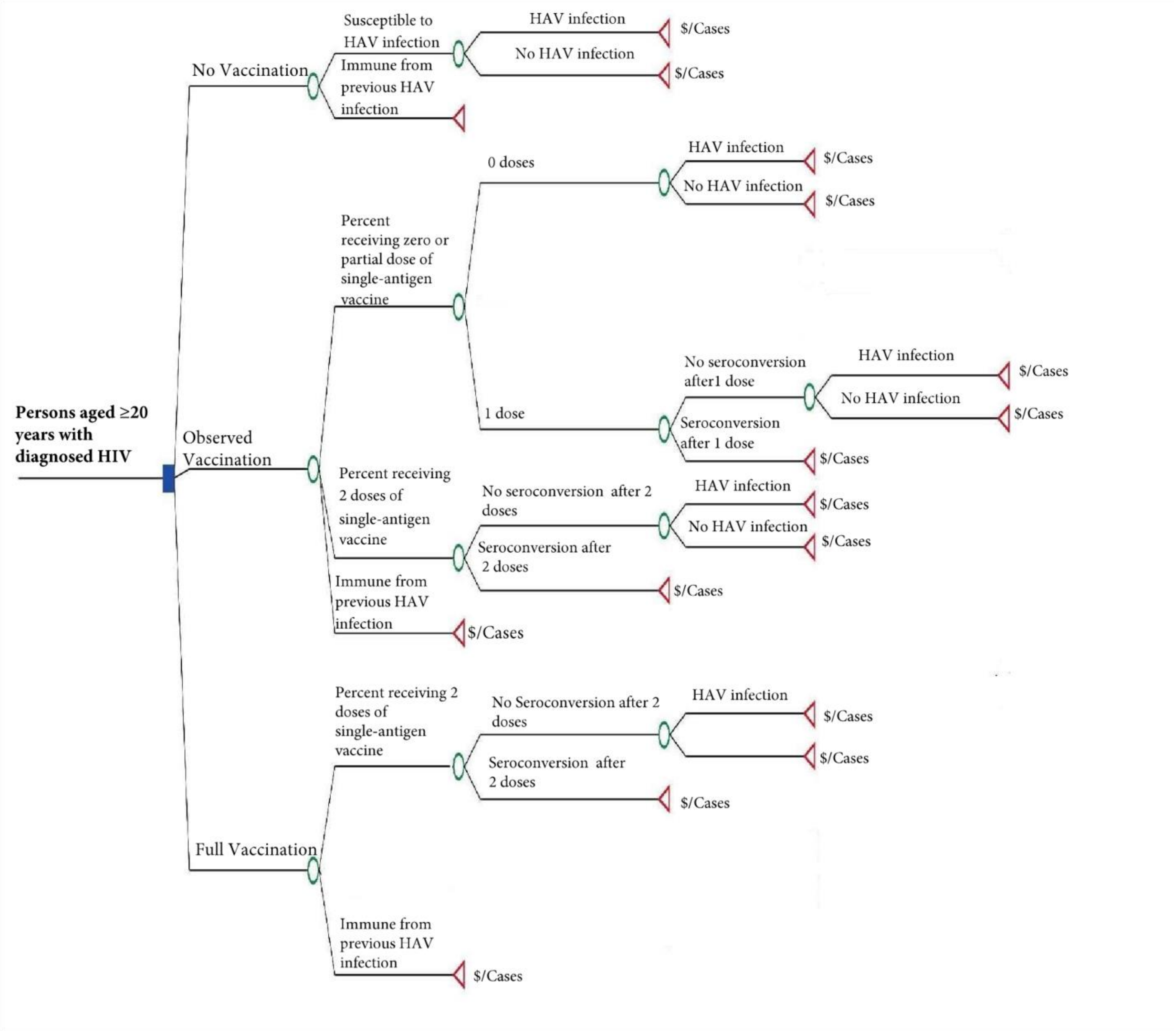

Supplemental Figure 1: Model structure - Single-antigen vaccine
