# Supplementary figures and images for: Cost-effectiveness of expanded hepatitis A vaccination among adults with diagnosed HIV, United States
Source: PLoS One. 2023 Mar 17;18(3):e0282972. doi: 10.1371/journal.pone.0282972 (PMC10022807; doi:10.1371/journal.pone.0282972)

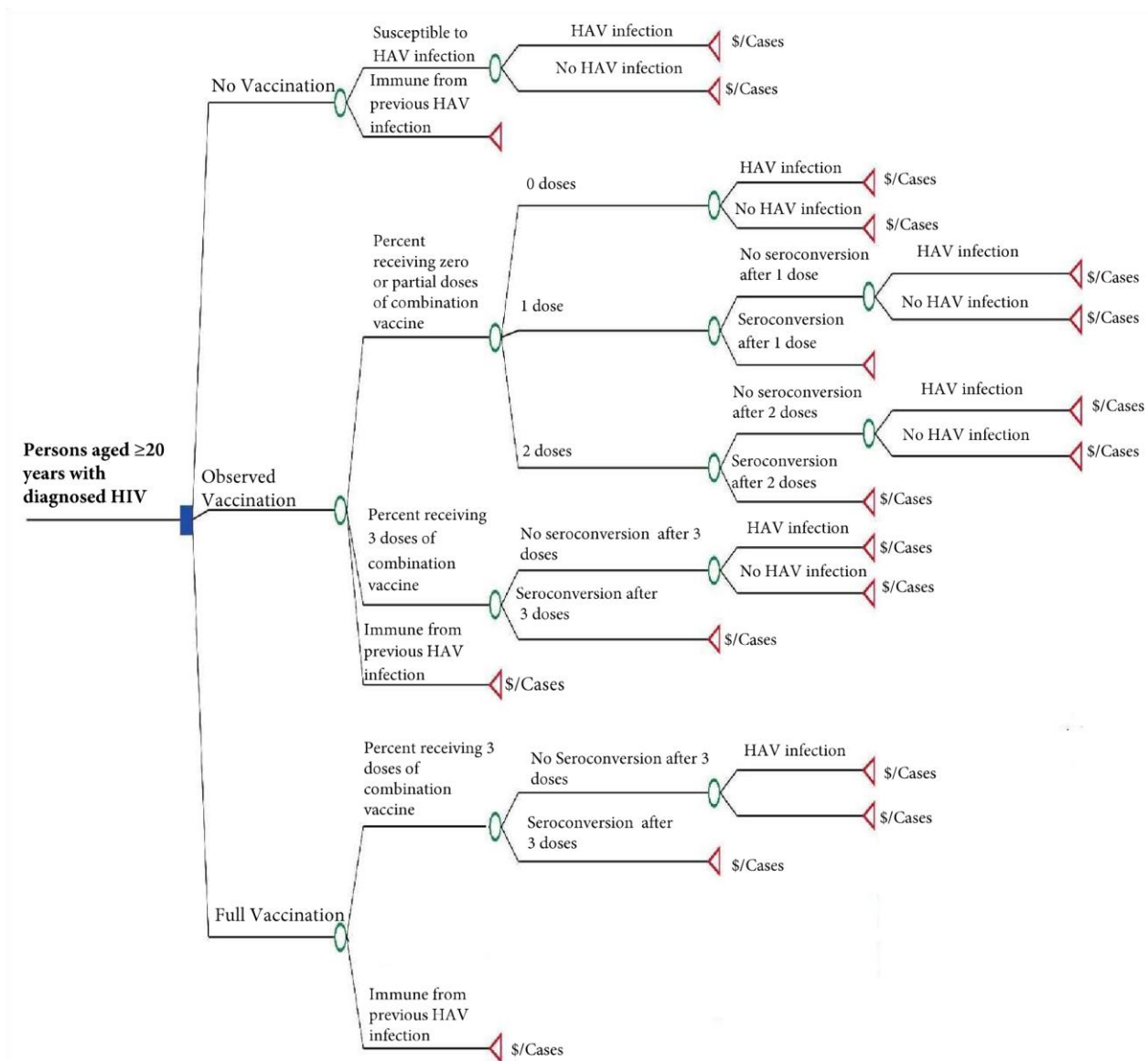

**Supplemental Figure 2: Model structure - combination vaccine**

Supplement: S2 Fig — This figure shows the model structure or schematic developed in TreeAge pro for the dual antigen vaccine. (PDF) [file pone.0282972.s002.pdf]
